# Tuning properties of biomimetic magnetic nanoparticles by combining magnetosome associated proteins

**DOI:** 10.1038/s41598-019-45219-7

**Published:** 2019-06-19

**Authors:** Ana Peigneux, Ylenia Jabalera, Ma Antonia Fernández Vivas, Salvador Casares, Ana I. Azuaga, Concepción Jimenez-Lopez

**Affiliations:** 1 Department of Microbiology, University of Granada, Campus de Fuentenueva s/n, 18071 Granada, Spain; 2 Department of Physical Chemistry, University of Granada, Campus de Fuentenueva s/n, 18071 Granada, Spain

**Keywords:** Bioinspired materials, Biomineralization, Biomimetic synthesis

## Abstract

The role of magnetosome associated proteins on the *in vitro* synthesis of magnetite nanoparticles has gained interest, both to obtain a better understanding of the magnetosome biomineralization process and to be able to produce novel magnetosome-like biomimetic nanoparticles. Up to now, only one recombinant protein has been used at the time to *in vitro* form biomimetic magnetite precipitates, being that a scenario far enough from what probably occurs in the magnetosome. In the present study, both Mms6 and MamC from *Magnetococcus marinus* MC-1 have been used to *in vitro* form biomimetic magnetites. Our results show that MamC and Mms6 have different, but complementary, effects on *in vitro* magnetite nucleation and growth. MamC seems to control the kinetics of magnetite nucleation while Mms6 seems to preferably control the kinetics for crystal growth. Our results from the present study also indicate that it is possible to combine both proteins to tune the properties of the resulting biomimetic magnetites. In particular, by changing the relative ratio of these proteins, better faceted and/or larger magnetite crystals with, consequently, different magnetic moment per particle could be obtained. This study provides with tools to obtain new biomimetic nanoparticles with a potential utility for biotechnological applications.

## Introduction

Magnetotactic bacteria form an ubiquitous and heterogeneous group of prokaryotic microorganisms that possess an unique organelle, the magnetosome, formed by a magnetic mineral [magnetite (Fe_3_O_4_) or greigite (Fe_2_S_4_)] surrounded by a lipid bilayer^1–4^. Magnetosomes constitute the ideal magnetic nanoparticles^5^ that could be used in numerous nanotechnological applications in which they show important advantages over other type of nanoparticles. Among these applications are the detection of nucleotidic polymorphism^6,7^, cell separation^8^, DNA isolation and purification^9^, contrast agent in magnetic resonance imaging [MRI]^10^, early diagnosis, drug transporter/carrier for a targeted chemotherapeutic treatment^11^ and hyperthermia cancer treatments, understanding by that thermal damage induced by dipolar magnetic interactions^12–14^.

However, the massive production of magnetosomes cannot be done up to date because of the difficulties scaling up the culture of magnetotactic bacteria, being that the bottleneck for the application of magnetosomes in nanotechnology. In this context, one of the proposed alternatives to *in vitro* produce magnetosome-like magnetic nanoparticles without the need of cultivating magnetotactic bacteria is biomimetic, i.e the *in vitro* production of magnetosome-like magnetic nanoparticle mediated by magnetosome associated proteins (MAPs), which are crucial for the *in vivo* magnetosome formation^5,15–17^. The ability of some of these MAPs, expressed as recombinant proteins, to *in vitro* control magnetite nucleation and/or crystal growth has been showed by several authors^18–30^. In fact, magnetite crystals formed *in vitro* in the presence of these proteins are distinct to those formed in their absence under identical conditions and they present some magnetosome-like features. Mms6, MamD (Mms7), MamC (Mms13), MamG (Mms5) and MmsF are MAPs already identified as candidates to *in vitro* produce biomimetic magnetic nanoparticles (BMNPs)^15,16,31,32^. In this context, much work has been done by using Mms6 (either full length expressed as recombinant protein or synthetic peptides) from *Magnetospirillum magneticum* AMB-1^18–22,24,25,27,28^. MamC from *Magnetococcus marinus* MC-1^26,29,33^ and MmsF from *Magnetospirillum magneticum* AMB-1^31^ have also been studied, although in a much less extent. Therefore, and although those proteins have been showed to individually control the size and/or the morphology of the resulting magnetite precipitated *in vitro*, only one protein was introduced at the time in the reaction mixture from which magnetite was precipitating and the combined effect of introducing mixtures of them in the same precipitation reaction has not been yet tested. Combining different proteins in *in vitro* experiments creates an scenario probably closer to that of the magnetosome in which there are high chances that several magnetosome proteins are simultaneously involved in the nucleation and growth of the magnetite crystals. Therefore, it may open new ways to tune some properties like size, morphology and, consequently, magnetic moment of the resulting biomimetic nanoparticles. Consequently, the goal of this paper is to determine the effect of introducing two MAPs, Mms6 and MamC from *Magnetococcus marinus* MC-1, expressed as full length recombinant proteins, at different ratios in the reaction mixture from which magnetite precipitates, being the first time in which two proteins from the same magnetotactic bacteria have been introduced in the same reaction mixture.

MamC was chosen because it is the second most abundant protein in the known magnetotactic bacteria^34,35^ and, moreover, its effect on the size and shape on magnetite crystals grown *in vitro* in the presence of this protein was previously demonstrated^26,29,30^. Mms6 has been identified as the most abundant magnetosome protein^35^ and, moreover, Mms6 from AMB-1 has been extensively used in *in vitro* magnetite precipitation experiments by several groups^18–22,24,25,27,28^. However, this is the first time that Mms6 from *Magnetococcus marinus* MC-1 is expressed as recombinant protein, purified and used to *in vitro* precipitate magnetite.

## Results

### *In silico* analysis of the magnetosome associated protein Mms6

**Figure 1:**
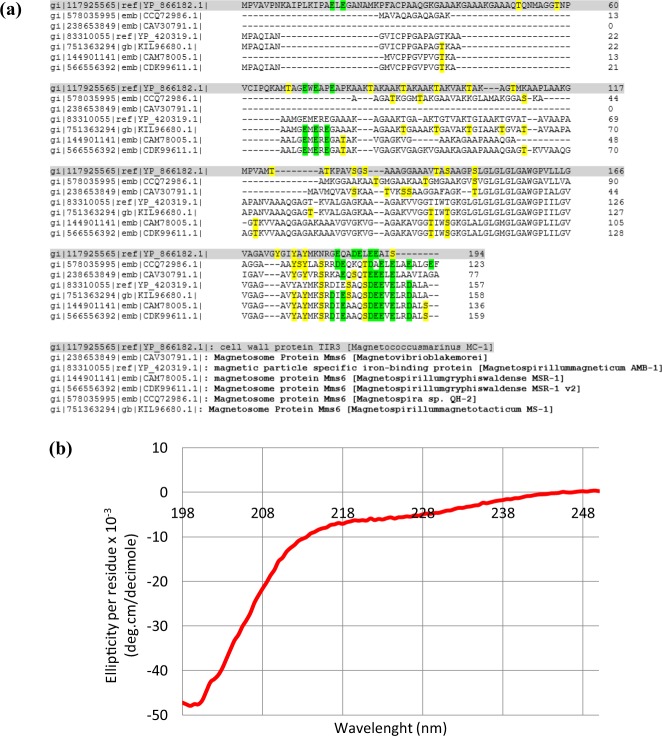
(**a**) CLUSTAL O (1.2.1) multiple sequence alignment of Mms6 protein in different magnetotactic bacteria. Negatively charged amino acids (Asp, Glu) are marked in green and amino acids containing hydroxyl groups (Tyr, Thr, Ser) in yellow. (**b**) Circular dichroism (CD) spectra in the far-UV (190–250 nm) of Mms6 protein.

Being the first time that Mms6 from MC-1 was purified, a multiple sequence alignment of this Mms6 with other homologous proteins from other magnetotactic bacteria was done for comparison. The analyses show similarities in the C-terminal domain of all Mms6 homologous proteins compared (Fig. 1a). This C-terminal is rich in acidic amino acids (aspartate and glutamate) and in amino acids containing hydroxyl groups (tyrosine, threonine, and serine), which can bind metal cations. As in the other Mms6 homologous, Mms6 from MC-1 has one predicted transmembrane region, and a high grade of hydrophobicity due to the transmembrane α-helix and the N-terminal region (Fig. S1a).

### Purification of MamC and Mms6 proteins and characterization of Mms6

SDS-polyacrylamide electrophoresis (SDS-PAGE) of MamC and Mms6 show intense bands with a high grade of purity (>90%) and a migration pattern corresponding to the theoretical molecular weight values calculated (17.46 and 22.5 KDa for MamC-His and Mms6-His, respectively; Fig. S1b). Figures S2a and S2b show the identity of Mms6 protein confirmed by peptide mass fingerprinting (PMF) (Fig. S2a) and peptide fragmentation (PFF) (Fig. S2b) by MALDI-TOF/TOF. The CD spectrum presents a minimum at 197 nm, which is characteristic of proteins with high random coil or unstructured content (Fig. 1b).

### Analysis of Mms6-MamC mediated magnetite nanoparticles

**Figure 2:**
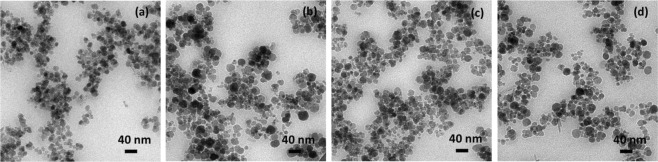
TEM images of magnetite formed in: (**a**) MamC-buffer experiments, (**b**) Mms6-buffer experiments, (**c**) empty-vector experiments and (**d**) inorganic (protein free) experiments.

The solids formed in all the biomineralization experiments were identified as magnetite using XRD. TEM analysis of the magnetite particles produced in MamC-buffer experiment (Fig. 2a), Mms6-buffer experiment (Fig. 2b), empty-vector experiment (Fig. 2c) and inorganic control experiment (Fig. 2d) show similar crystal sizes of 16 ± 6 nm. Also, no differences in morphology were observed either, being the particles poorly faceted. Therefore, the potential differences on the crystal size and/or morphology observed on the magnetites collected from the protein-bearing experiments should be solely attributed to the proteins involved.

**Figure 3:**
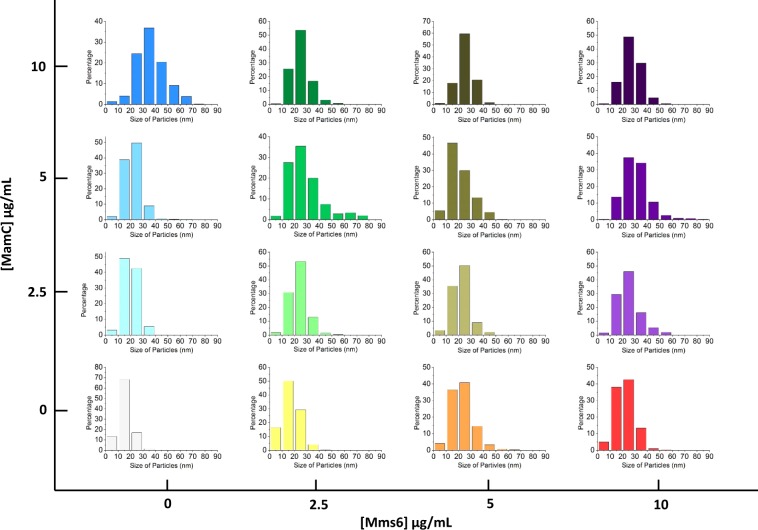
Size distribution histograms of particles obtained in MamC-experiments, Mms6-experiments and Mms6-MamC-experiments.

TEM images of the Mms6-mediated magnetites show differences in size and shape with respect to those from the inorganic control experiments, depending on the concentration of Mms6 in solution (Fig. S3). At Mms6 concentration of 2.5 µg/mL, non-faceted crystals of 17 ± 7 nm similar to those from the control experiments precipitated from solution. However, at Mms6 concentrations of 5 and 10 µg/mL, magnetite crystals had uniform polyhedral morphologies with well-faceted faces and sizes of ∼23 nm, which are significant larger than those of magnetites obtained from the inorganic control experiment (Figs 3, S3 and S4a). By adjusting the average size of the magnetite crystals versus the relevant protein concentration (Fig. S4b,c), regression lines and slopes were determined. The size of the magnetite crystals formed in the presence of solely Mms6 increased at a rate of 1.58 nm per µg/mL of Mms6 (R^2^ = 0.6803) up to [Mms6] = 10 µg/mL (Fig. S4b). In the context of MamC, magnetite crystals formed in the sole presence of 2.5 µg/mL and 5 µg/mL of this protein had sizes of 20 ± 6 nm and 22 ± 7 nm, respectively. At 10 µg/mL of MamC, magnetite crystals displayed well-developed crystal faces and sizes of 37 ± 12 nm (Figs 3, S3 and S4a). In this case, the rate of the increase of the size of the crystal with respect to the concentration of MamC was 4.42 nm per µg/mL (R^2^ = 0.799) (Fig. S4c).

When both MamC and Mms6 were present in the reaction solution, cumulative effects from both proteins were observed, since, as it was shown, crystals obtained in the presence of both proteins were different in size and/or morphology than those obtained in the presence of each one of these proteins separately or if no protein was present (Figs 3, S3 and S4a). In fact, magnetite crystals collected from these experiments displayed better faceted morphologies and/or larger sizes compared, not only to crystals from the inorganic control experiment, but also to crystals collected from experiments in which only one of the proteins was present. At low concentrations of Mms6 (2.5 µg/mL), the size of the crystals increased with the concentration of MamC up to [MamC] = 5 µg/mL (Figs 3 and S4b,c). This trend is identical to that observed at the highest concentration of Mms6 (10 µg/mL). However, at [Mms6] = 5 µg/mL no change in the size of the crystals was observed independently of the concentration of MamC in the solution. The larger crystals are obtained at MamC concentration of 5 µg/mL + Mms6 concentration of 10 µg/mL. At higher MamC/Mms6 ratios, crystal size decreases, being this decrease statistically significant (Table S2). It is interesting to notice that the averages for crystal sizes obtained by introducing only MamC in the reaction mixture at a given concentration are always (with the exception of comparing [MamC] and [Mms6] at 5 µg/mL) statistically different than those obtained when Mms6 is individually introduced at identical concentration or the mixture of MamC + Mms6 at a total protein concentration that matches that of the individual MamC (Table S2). This result seems to indicate that MamC and Mms6 affect the nucleation and growth processes differentially.

**Figure 4:**
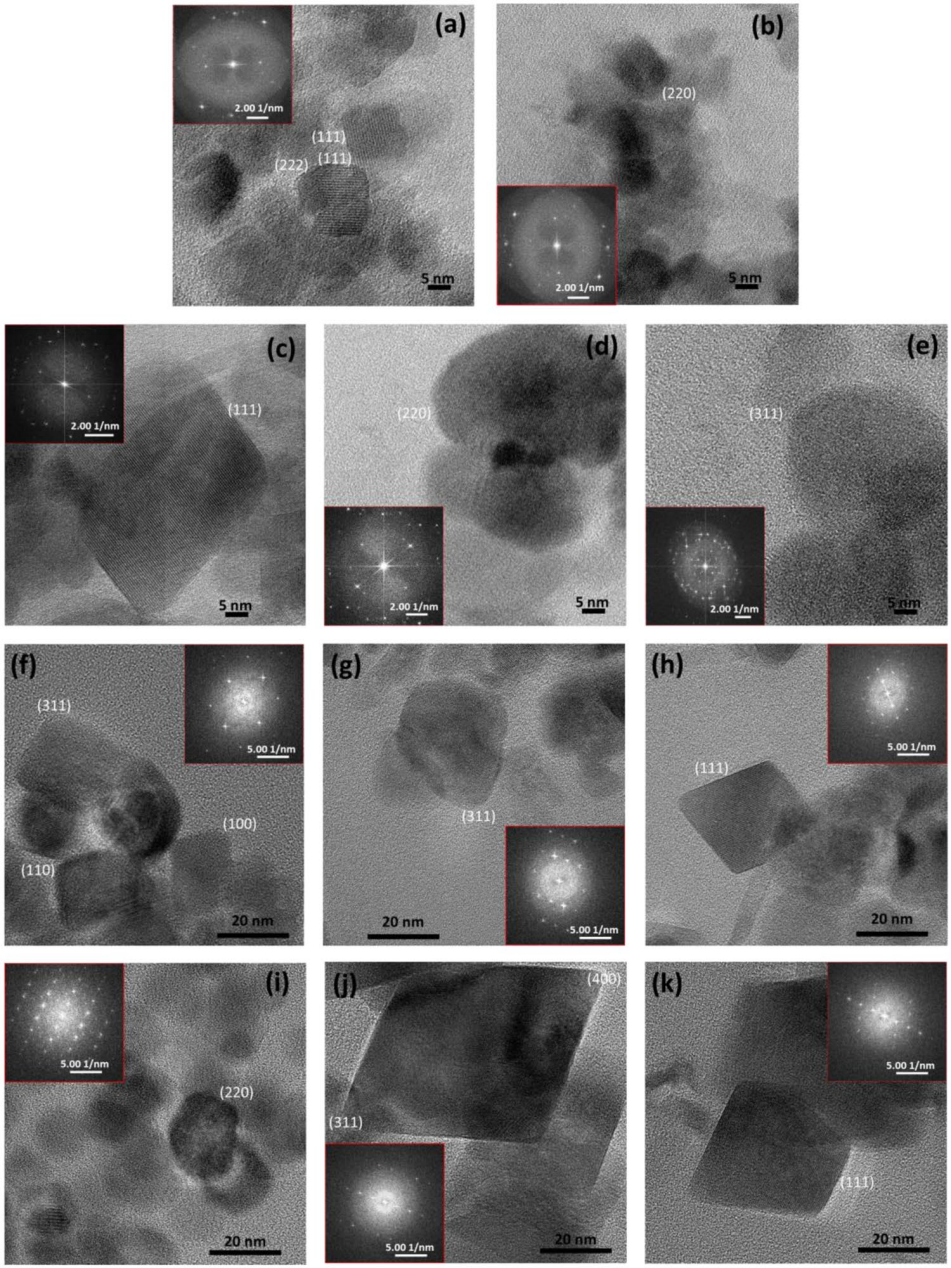
HRTEM images of (**a**,**b**) inorganic magnetite nanoparticles, (**c**–**e**) MamC-magnetite nanoparticles, (**f**–**h**) Mms6-magnetite nanoparticles, and (**i**–**k**) Mms6-MamC-mediated nanoparticles. Selected areas electron diffraction are shown for each sample.

HRTEM images show that crystals obtained from the inorganic control experiments have a square and rhombic 2-D shapes bounded by (111) face and a few crystals showed rounded corners corresponding to incipient (110) crystal face (Fig. 4a,b). MamC-mediated nanoparticles expressed the (111) crystal face with rounded corners corresponding to nascent (110) and (311) crystal faces (Fig. 4c–e). In this case, crystals appeared elongated along [111] direction. Crystals obtained in the presence of Mms6 protein also showed rhombic, rectangle and hexagon shapes bounded by (111) crystal face and rounded corners corresponding to (311), (110) and (100) crystal faces (Fig. 4f–h). These crystals were elongated as well along the [111] direction. Nanoparticles obtained at 5 μg/mL of MamC and 10 μg/mL of Mms6 expressed the same faces listed above [(111), (110), (311) and (100)], crystals also elongated along [111], but in this case shapes and corners were the best defined of all experiments.

**Figure 5:**
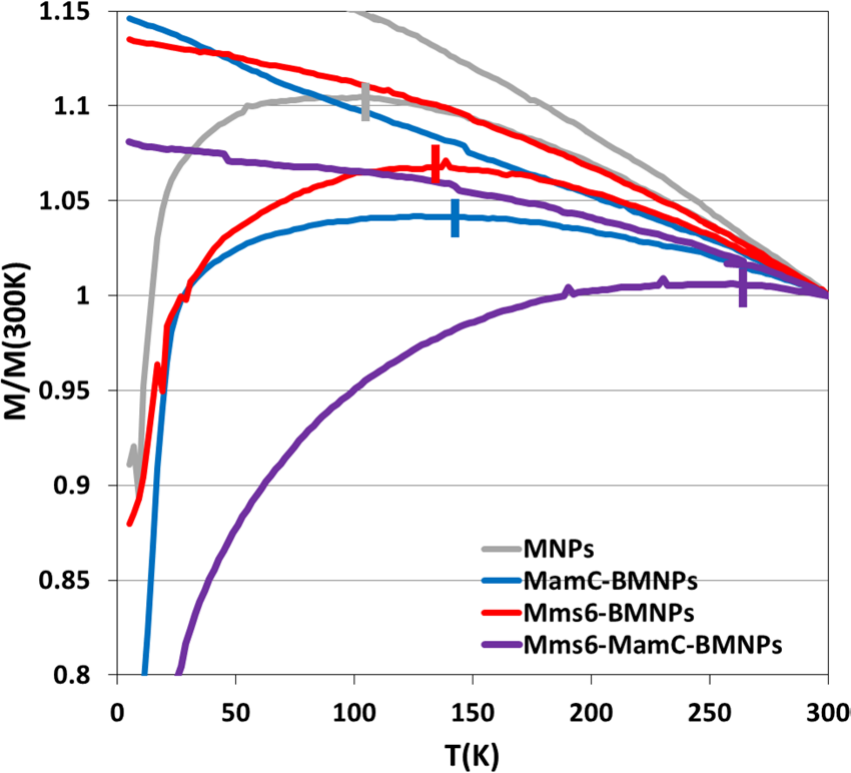
ZFC-W and FC-C of inorganic magnetites (MNPs), MamC mediated biomimetic nanoparticles (MamC-BMNPs), Mms6 mediated biomimetic nanoparticles (Mms6-BMNPs) and Mms6 + MamC mediated biomimetic nanoparticles (Mms6-MamC-BMNPs).

ZFC-FC curves at 500 Oe show differences between the different biomimetic and inorganic magnetic nanoparticles (Fig. 5). The slowest increase in magnetization was found in Mms6-MamC-BMNPs while the faster increase occurred in the inorganic (protein-free) experiments (MNPs). Moreover, the blocking temperature and the irreversibility temperature of the different biomimetic particles and those of MNPs are also different. The lowest T_B_ (103 K) and T_irr_ (274 K) correspond to MNPs, then to Mms6-BMNPs, then MamC-BMNPs while the largest T_B_ (260 K) and T_irr_ (296 K) correspond to Mms6-MamC-BMNPs. This slow magnetization increase and higher T_B_ and T_irr_ is consistent with particles with high crystallinity and a large magnetic moment per particle, also consistent with a less polydisperse magnetic moment^5^.

## Discussion

It seems clear from these results that both MamC and Mms6 alter the kinetics of magnetite nucleation and growth. In fact, the size of the magnetite crystals formed in the presence of either each protein individually, or in the presence of a combination of both proteins, is significantly larger than that of the crystals obtained in the absence of any protein and, also, the combination of both proteins results in magnetite crystals different in size and/or morphology than those formed in the presence on any of these proteins individually. Moreover, both proteins affect such a kinetic differentially, since the presence of MamC has a greater effect on the size of the crystals compared to that of Mms6. This output was also observed by Nudelman *et al*.^36^.

In the context of the individual effect of each protein on the *in vitro* magnetite nucleation and growth, Nudelman and Zarivach^15^ predicted that the secondary structure of Mms6 from *Magnetospirillum gryphiswaldense* MSR-1 shows an unstructured N-terminal with a transmembrane region and an acidic C-terminal, which may form an α-helix structure, which is exposed to the magnetosome lumen and, thus, it would be able to interact with the magnetite crystal. In fact, several studies have demonstrated that the C-terminal of Mms6 from AMB-1 controls the size and morphology of *in vitro* synthesized magnetite^19,22,28,37^. Acidic amino acids [Asp123, Glu124, Glu125^38^] are claimed to be responsible for such a control through iron binding. Some of these amino acids (Asp12 and Glu13) have been identified in Mms6-MIC as strong iron binders with a low dissociation constants (Kd)^36^. Although these studies have been performed with Mms6 proteins from *Magnetospirillum magnetotacticum* AMB-1^18–22,24,25,27,28^, our multiple sequence analysis show that this C-terminal is relatively conserved into different species (Fig. 1a). In fact, in the context of MC-1, the C-terminal of Mms6 has 3 tyrosines, 4 glutamic acids, 1 aspartic acid, and 1 serine that can bind metal cations^39^. These acidic amino acids (glutamic and aspartic acids) are present in the C-terminal of all the sequenced Mms6 proteins, only varying the number of them (between 5 and 7) among the different homologous proteins (Fig. 1a). In this case, the percentage of negatively charged amino acids is 17.9% of the total amino acids of the C-terminal and, as proposed by previous authors, those acidic amino acids may bind Fe cation, being Asp12, Glu13, Glu16, and Asp19 (in Mms6-MIC peptide) specially relevant in terms of Kd (0.21 mM ± 0.12 mM for Fe^2+^)^36^. Such binding results in a local increase of the supersaturation of the system with respect to magnetite and, thus, the nucleation of such a phase is induced in those specific areas due to an ionotropic effect^18,19,22,28,33,37,38,40^.

Different is the case of MamC, as not only an ionotropic effect, but also a template effect have been claimed to explain the role of this protein in the nucleation and growth of magnetite *in vitro*. On one hand, MamC from MC-1 contains 5 amino acids negatively charged that represent 15.6% of the total amino acids of the loop. Moreover, Nudelman *et al*.^36^ identified in MamC-MIC two residues with special affinity for Fe^2+^: Asp14, which corresponds to the Asp70 of the full length protein from AMB-1 (NCBI reference: WP_011383388.1), a residue that was already known to play a role in magnetite nucleation and binding^29^, and Gly16. Therefore, as in the case of Mms6, ionotropic effects could induce magnetite nucleation on those specific negatively charged areas. However, some authors^29,30,41^ also claim a template effect that rules magnetite nucleation based on: a) the distance between the Glu66 and Asp70 (8 Å) is similar (within the helix elasticity) to the 6 Å distance between the Fe cations in specific crystal faces, namely (111), (100), (110) and (311), that become expressed in the final morphology of MamC-mediated magnetites, and b) when MamC was not correctly folded, the size of the resulting crystals was equal to that from crystals precipitated in protein-free experiments, so an extended protein structure was needed for MamC to control the size and/or morphology of magnetite.

In the protein-free experiments, and since the system is supersaturated with respect to magnetite [logΩ_magnetite_ = 22.57^26^] bulk nucleation occurs, giving rise to the formation of a large number of crystals with small size, probably being the restricted concentration of Fe cation the limiting step for crystal growth. Different is the scenario when the proteins are present, since, by providing nucleation sites, magnetite nucleation is kinetically favored with respect to bulk nucleation (either due to the ionotropic and/or template effects)^42^ and, therefore, less nuclei form than can grow to larger sizes compared to those formed in the protein-free experiment. However, and since the concentration of Fe in solution is limited, such a phenomenon could be reversed if the number of nucleation sites is too large, so, at some point, those nuclei cannot grow any further because there is no more Fe available in solution. This explains why magnetite crystals produced in the experiments containing the highest protein concentrations (especially MamC) were smaller than those grew at lower protein concentrations (Figs 3, S3 and S4a). These results are in agreement with the trend observed by Valverde-Tercedor *et al*.^26^.

Our observations that MamC and Mms6 affect crystal size and morphology differently have previously being observed also by Nudelman *et al*.^36^ working with MamC and Mms6 peptides (MamC-MIC and Mms6-MIC). These authors observed that the MamC-MIC showed the weakest binding of ions but, however, created the most significant effect in enhancing magnetite particle size. Instead, the strong ion binder Mms6-MIC had almost no effect in modulating magnetite particle size. These authors concluded that the strong ion-binding affinity of Mms6 might be critical for nucleation by ionotropic effect while MamC mainly contributes modulating magnetite particle size and shape and potentially recognizing particles. Therefore, these authors suggest that the regulation of magnetite particle formation and the recruiting of metal ion could be decoupled.

Our interpretations fall along this suggestion with some modifications. According to the results of Bereczk-Tompa *et al*.^43^ and being the iron binding by negatively charged amino acids (in extended surfaces) a less specific process than the binding of previously formed nuclei, magnetite nucleation induced by the ionotropic effect is probably kinetically favoured in the case of MamC^44^. HRTEM images of MamC-mediated magnetite crystals in Lopez-Moreno *et al*.^30^ showing that they are single crystals with no discontinuities in the crystal lattices also supports this hypothesis versus the oriented aggregation of previously formed nuclei.

The question then remains what effect is kinetically more favourable for nucleation under the conditions of the present study, whether it is template effect (MamC) or ionotropic effect (mainly Mms6 but also MamC in a less extent). Under the limited iron conditions in which our experiments were run, if Mms6 was controlling nucleation by ionotropic effect, then, a given concentration of this protein would determine the number of nuclei that form and, in turn, being the iron concentration in the solution limited, the size of the crystal would be inversely related to the number of nuclei. As a consequence, Mms6 should have had an important role controlling the size of the crystal. Conversely, it was MamC, and not Mms6, the protein that had more input determining the size of the crystals. Several studies^26,29,30,41,44,45^ have shown the effect of MamC on the size of the magnetite crystals formed in the presence of the protein and had demonstrated the importance of the conformation of MamC loop in properly controlling such a size. Therefore, the template effect, rather than the ionotropic effect seems to stand as the key factor controlling nucleation and the size of the final crystals under limited iron conditions.

Once formed, these nuclei grow, first probably at the expenses of the Fe available in the bulk solution, and then, from the Fe previously bind by the acidic amino acids in the proteins, which act as Fe reservoirs for crystal growth. The fact that better faceted crystals are obtained when Mms6 is present in the solution supports this hypothesis, indicating that the effect of Mms6 is mainly directed to lower the kinetics of crystal growth by lowering the supersaturation of the system with respect to magnetite.

As a summary, our model of how magnetite nucleation and growth occurs in the presence of MamC and Mms6 is the following: a) First, acidic amino acids of Mms6 and MamC bind Fe cations from the solution by an ionotropic effect, also confirmed by the results of Nudelman *et al*.^36^, thus lowering the supersaturation of the system with respect to magnetite and preventing bulk nucleation. Then, nucleation occurs in the extended surface provided by the MamC loop mainly driven by a template effect. MamC strongly controls the kinetic of nucleation determining the number of nuclei that form, which would be dependent on the concentration of MamC with the adequate conformation. b) Secondly, previously formed nuclei grow at the expense of the Fe cations in solution and then, when needed for crystal growth, the Fe cations concentrated at the acidic amino acids of, firstly, MamC and, then, Mms6 (according to the Kd calculated by Nudelman *et al*.^36^ are released and become available. Mms6 thus controls the kinetics of crystal growth since the binding of Fe and the controlled release of such cations lowers the supersaturation of the system at which magnetite grows. c) Magnetite crystal grow slower, such a growth being controlled by the release of the Fe cations from Mms6, thus, the resulting magnetite crystals accumulate a lower number of defects. d) This growth occurs while the system is supersaturated with respect to magnetite, probably being Fe cation the limitant component for further crystal growth.

Some of the crystal faces identified for Mms6 in the present work were previously described by other authors in Mms6 (from AMB-1)-mediated magnetites synthesized *in vitro*. For instance, Arakaki *et al*.^22^ observed the expression of the crystal faces (400) and (311). Also Amemiya *et al*.^19^ observed the expression of the (100) face. Curiously, and although we are aware that the direct extrapolation of the results obtained *in vitro* to the *in vivo* scenario is not possible, the crystals faces observed in the present study (Mms6-MamC-magnetites) are identical to those determined by Mann *et al*.^46^ for MC-1 magnetosomes.

For that combined effect to happen, MamC and Mms6 may or may not have to be physically interacting by an specific (or inespecific) interaction, as long as the Mms6 C-terminal and the MamC-loop are intact. Preliminary immuno-precipitation experiments carried out in our laboratory with these two proteins (Fig. S5) gave us some hints suggesting the existence of some type of interaction. However, in order to unambiguously prove this specific interaction, further experiments are needed that should be directed not only to prove the interaction, but also to determine de area of both proteins involved in it. This would not be the first case in which interactions between magnetosome proteins have been described^34,47^. For example, the interaction of MamK and MamJ, essentials to the assembly of magnetosomes in a chain, was determined by Carrilo *et al*.^48^. Up to date, there is only experimental evidence for the interaction between Mms6 (from *M. magneticum* AMB-1) and MamA^49^. MamA is not directly involved in magnetite formation, but it has TPR motifs that are known to play an important role in protein–protein interactions^15,32^. Also, Tanaka *et al*.^50^ proposed that the N-terminal of Mms6 could have sorting functions to properly localize other proteins onto the magnetite crystal surface.

The larger and better crystals obtained in presence of both MamC and Mms6 were consistent with the measurements of the magnetic moment of the solid samples. While the size of the particles in all cases falls within the range of single magnetic domain^5^, the blocking temperature of the particles (T_B_ < 300 K in all cases) indicates that they do not display a net magnetization in the absence of an external magnetic field probably because the crystals have their magnetic moments randomly distributed at such temperature^5^. The slower magnetization increase and higher T_B_ are characteristic of particles of higher crystallinity, larger size, and a larger magnetic moment per particle^5^. In this context, all biomimetic magnetic nanoparticles display higher T_B_ compared to that of MNPs. This is important since for clinical applications, in the absence of an external magnetic field, the samples do not show magnetization, probably caused by a random distribution of magnetic moments at this temperature, which would prevent agglomeration. However, once an external magnetic field is applied in order to direct the nanoparticles to the target site, the larger the magnetic moment per particle is, the more efficient response is expected. Both crystal size and crystallinity accounts for this difference in T_B_. Therefore, the possibility of producing magnetic nanoparticles with tuned magnetic properties by combining recombinant magnetosome proteins has an important potential in the design of magnetic nanoparticles for biotechnological applications.

## Conclusions

The present study demonstrates that it is possible to combine MamC and Mms6 proteins from *M. marinus* MC-1 to obtain *in vitro* biomimetic magnetite nanoparticles different than those obtained by using only one of the proteins at a time and/or no protein at all. The combined effect of MamC and Mms6, specifically at MamC concentrations of 5 µg/mL and Mms6 concentrations of 10 µg/mL, produces *in vitro* well faceted crystals both large in size (30 ± 10 nm) and with the highest blocking temperature, indicating the largest magnetic moment per particle. Although the presence of MamC and Mms6 affects magnetite nucleation and growth *in vitro*, they affect the kinetics of both processes differently. MamC seems to control the kinetics of crystal nucleation because of the combined ionotropic and template effects while Mms6 seems to preferably control the kinetics of crystal growth by acting as an Fe reservoir. These experiments provide with novel biomimetic magnetic nanoparticles that could be potentially useful in nanotechnological applications.

## Methods

### Cloning, expression and purification of recombinant MamC

*Magnetococcus marinus* MC-1 cells were grown microaerobically under chemolithoautotrophic conditions with thiosulfate as the electron donor in cultures as described by Williams *et al*.^51^. Genomic DNA from *Magnetococcus marinus* MC-1 #ATCC BAA-1437(T), JCM 17883(T) was isolated following the method described by Martín-Platero *et al*.^52^. MamC cloning, expression and purification were carried out as described in Valverde-Tercedor *et al*.^26^. Briefly, the *mamC* gene was cloned into a pTrcHis-TOPO vector (Life Technologies: Invitrogen, Grand Island, NY) so that the recombinant MamC protein is expressed with an N-terminal hexahistidine tag. The recombinant vector was transformed into an *Escherichia coli* TOP10 strain (Life Technologies: Invitrogen) and MamC expression was induced with isopropyl β-D-1-thiogalactopyranoside (IPTG). A HiTrap chelating HP column (GE Healthcare) was used for protein purification under denaturing conditions and MamC was later folded by sequential removal of the urea initially contained in the elution buffer.

### *In silico* analysis of Mms6. Cloning, expression and purification of recombinant Mms6

Sequence alignments of Mms6 protein with homologous proteins in other bacteria were performed using Clustal Omega. All amino acid sequences of those proteins were obtained from NCBI Database. Hydrophobicity and physicochemical properties of Mms6 were deduced from its protein sequence using the ExPAsy Server. The *mms6* gene from *M. marinus* MC-1 (NCBI Database, gene accession ABK44776.1, protein accession Mmc1_2275) was amplified by polymerase chain reaction using the specific primers: f6 (SEQ ID NO: 1, 5′-ATGCCTGTTGCTGTACCAAATAAAGC-3′) and r6 (SEQ ID NO: 2, 5′-TCAGCTAATGGCCTCTTCCAATTC-3′). The amplified *mms6* gene was cloned into a pTrcHis-TOPO vector and the host was *E. coli* TOP10. The amplified gene was verified by dideoxynucleotide sequencing. The expression of Mms6 was almost identical to that of MamC, but 1 mM IPTG was used instead. After centrifugation cells were resuspended in 20 mM sodium phosphate buffer (pH 7.4) supplemented with 0.5 mg/mL lysozyme and 5% sodium lauroylsarcosinate (sarkosyl) and disrupted by sonication. The soluble fraction was separated by centrifugation and loaded onto a HiTrap chelating HP column (GE Healthcare) by using an ÄKTA Prime Plus FPLC System (GE Healthcare). The column was previously equilibrated with 20 mM sodium phosphate buffer (pH 7.4) supplemented with 20 mM imidazole and Triton X-100 at 1.3 x the critical micelle concentration (CMC) to reduce protein aggregation and to improve protein stability. The elution of Mms6 (2 mL/min) was performed by applying a continuous imidazole gradient from 20 to 500 mM. Fractions were collected and analyzed by 12% SDS-PAGE electrophoresis. Fractions containing Mms6 were subjected to an additional chromatographic step in a C4 hydrophobicity column (Jupiter® 5 µm C4 300 Å, LC Column 150 × 4.6 mm) using a HPLC system (Agilent 1100) to remove minor contaminants, *E. coli* proteins, and nucleic acids. In this case, the elution of Mms6 protein (0.5 mL/min) occurred by applying a continuous organic solvent (trifluoroacetic acid and acetonitrile) gradient into water because of the high hydrophobicity of Mms6. The purity of the Mms6 protein was tested by Coomassie-stained 12% SDS-PAGE. Protein concentration was determined using a Bradford protein assay^53^ and using a NanoDrop 2000 UV-Vis Spectrophotometer (Thermo Scientific), by using the corresponding molar extinction coefficient at 280 nm (17085 M^−1^ cm^−1^).

As a control experiment, TOP10 competent cells were also transformed with pTrcHis-TOPO that did not contain the genes of interest. The purification protocol of MamC and Mms6 was followed with those transformed bacteria and their corresponding elution fractions were used for magnetite precipitation (control) experiments.

Since MamC was extensively characterized by other authors^26,29,30,33^, all characterization analyses were performed on Mms6. This protein was analyzed by peptide mass fingerprinting (PMF) and peptide fragmentation (PFF) by MALDI-TOF/TOF. The protein was digested by reduction with dithiothreitol (DTT), derivatization with iodoacetamide (IAM) and subsequent digestion overnight with trypsin (45 ng) at 30 °C. The resulting peptides were extracted from the gel with 15 µl of 0.2% Trifluoroacetic acid (TFA) ad 30% acetonitrile. The samples were crystallized in an “AnchorChip” plate using α-Cyano-4-hydroxycinnamic acid (CHCA) as matrix, and analyzed in a MALDI TOF/TOF mass spectrometer (UltrafleXtreme, Bruker). The identification of the protein was carried out by using MASCOT 2.4.0 (MatrixScience) software^54^ as a search engine.

Circular dichroism (CD) spectra were recorded at 20 °C in the far-UV spectral region (190–250 nm) using a spectropolarimeter Jasco J-815 equipped with a Peltier-type cell holder. Measurements were performed in a 0.2 cm length × 1 mm band width quartz cell at a protein concentration between 0.2–0.8 mg/ml (time 1 s, scan rate 100 nm/min). Five consecutive scans were accumulated, and the average spectra were stored. Triton X-100 background was subtracted from protein spectra. Analysis of the experimental data was carried out with Spectra Manager software. Secondary structure composition was calculated by deconvolution of the spectrum using by Raussens *et al*. method^55^. The macromolecular structure of Mms6 protein suspensions deposited in carbon grids was analysed by transmission electron microscopy (TEM, LIBRA 120 PLUS Carl Zeiss SMT electron microscope).

### Biomineralization experiments

Deoxygenated solutions of NaHCO_3_/Na_2_CO_3_ (0.15 M/0.15 M), FeCl_3_ (1 M), Fe(ClO_4_)_2_ (0.5 M), and NaOH (5 M) were prepared by using oxygen-free deoxygenated Milli-Q water and magnetite precipitation was carried out in free-drift experiments held at 25 °C and 1 atm total pressure following the protocol described in Perez-Gonzalez *et al*.^56^. The final reaction mixture from which magnetite precipitated was 3.5 mM NaHCO_3_/3.5 mM Na_2_CO_3_, 2.78 mM Fe(ClO_4_)_2_, 5.56 mM FeCl_3_, pH = 9. MamC or/and Mms6 were added to the reaction mixture at concentrations ranging from 0 to 10 µg/mL. Specifically, nineteen magnetite coprecipitation experiments were carried out under the following conditions (three replica per condition) [1] sixteen experiments performed by adding MamC and Mms6 to the reaction solution at protein concentrations of 0, 2.5, 5, 7.5, 10 µg/mL and MamC/Mms6 and Mms6/MamC ratios ranging from 0 to 4, here referred as MamC-, Mms6-, Mms6-MamC-bearing experiments; [2] one experiment performed by adding to the reaction mixture the “contaminant” proteins purified from cells transformed with the “empty” pTrcHis-TOPO, here referred as empty-vector experiments; [3] two experiments carried out by adding to the reaction mixture the buffer in which each one of the proteins was stored (50 mM Tris and 150 mM NaCl, here referred as MamC-buffer experiments) and 1.3 CMC Triton X-100 in water (here referred as Mms6-buffer experiments); [4] one inorganic experiment in which no proteins and/or buffer were added to the reaction mixture. Experiments were always done in triplicate and particle size determinations were done for each one of the replica, all values accounting for the averages and standard deviations given for a particular experiment. Each experiment was allowed to proceed inside the anaerobic chamber for 30 days, after which the precipitated product was harvested. The solids were concentrated in tubes with a magnet and the supernatant (that looked completely clear) was discarded. Then the precipitates were washed with oxygen-free deoxygenated Milli-Q water two times and a last wash was performed with absolute ethanol (5 mL in each reaction). Between washes, each reaction flask was vigorously shaken for several seconds, the precipitate was magnetically concentrated, and the liquid removed. After the last ethanol washing the precipitate was concentrated in 1–2 mL of ethanol, hermetically sealed and stored at −20 °C until analyzed.

Powder samples of the precipitates were analyzed with an Xpert Pro X-ray diffractometer (PANalytical; The Netherlands) using the Cu Kα radiation, with the scan range set from 20 to 60° in 2θ (0.01°/step; 3 s per step). Identification of the precipitates was performed by using the XPowder software^57^. The morphology and size of the magnetite nanoparticles collected in those experiments were studied by Transmission Electron Microscopy (TEM) using a LIBRA 120 PLUS of Carl Zeiss SMT microcope. Magnetic nanoparticles were embedded in Embed 812 resin. Ultrathin sections (50–70 nm) were prepared using a Reichert Ultracut S microtome (Leica Microsystems GmbH, Wetzlar, Germany), after which the sections were deposited onto copper grids. The determination of the size of the crystals were done on TEM images taken at 20 kX magnification. These images were further maximized to whole screen, the scale bar was used to calibrate the pixel to nm conversion on the ImageJ 1.47 program and, then, the individual crystals were measured manually to avoid potential overestimation of the size due to particle aggregation, since it is well known than the human eye has a greater resolution compared to an image analysis program. To ensure reproducibility of results, crystal sizes were measured on several micrographs at 20 xK with an excess of 1000 nanoparticles for each experiment. The size distribution curves were determined from those measurements by using Origin pro 9. In addition, statistical significance of the results obtained was tested using Tukey test with a fixed value of α < 0.05. High Resolution TEM (HRTEM) was also performed at 500 Kx of magnification by using a FEI TITAN G2 80–300 and HRTEM Philips CM200 microscopes. The selected area electron diffraction (SAED) patterns were collected using a 10 µm aperture. D-spacings were measured using HRTEM images and the crystallographic direction was determined by using magnetite data in RRUFF Project Web site (http://rruff.info/ams/amcsd.php).

Zero-field cooling (ZFC-W) and field cooling (FC-C) measurements were carried out by using a superconducting quantum interference device (SQUID) 5 T magnetometer (Quantum Design MPMS XL, USA). Under gentle argon flow, a given amount of each specimen powder was placed in a double-walled polycarbonate capsule. The samples were immediately cooled in a zero applied field to 5 K to preserve randomized magnetization of the nanocrystals, after which a 500 Oe magnetic field was applied and samples were heated up to 300 K and then from 300 K without turning the field off. To allow comparison among the different complexes, the M(T) curves were normalized by the amount (g) of each sample analysed and by the magnetization value of the specific sample at 300 K. No distinction between the terms of “superparamagnetic” or “single magnetic domain” will be done in this work^5^. Blocking temperature (T_B_) was determined as that at which the maximum in magnetization occurred in ZFC curves, while irreversibility temperature (T_irr_) was such temperature that below the “blocking” of the superparamagnetic particles, which are no longer thermally equilibrated^5^.

## .Supplementary information

Supplementary information

Raw data Mms6 PMF spectrum

Raw data Mms6 MSMS spectrums
