# Supplementary material for: Tuning properties of biomimetic magnetic nanoparticles by combining magnetosome associated proteins
Source: Sci Rep. 2019 Jun 19;9:8804. doi: 10.1038/s41598-019-45219-7 (PMC6584501; doi:10.1038/s41598-019-45219-7)
Supplement: Supplementary file 1 — Supplementary information [file 41598_2019_45219_MOESM1_ESM.docx]

**Supplementary information**

**Tuning properties of biomimetic magnetic nanoparticles by combining magnetosome associated proteins**

Ana Peigneux^1^, Ylenia Jabalera^1^, Mª Antonia Fernández Vivas^1^, Salvador Casares^2^, Ana I. Azuaga^2^ and Concepción Jimenez-Lopez^1*^

^1^Department of Microbiology and ^2^Department of Physical Chemistry, University of Granada, Campus de Fuentenueva s/n, 18071 Granada, Spain,

*[cjl@ugr.es](mailto:cjl@ugr.es)


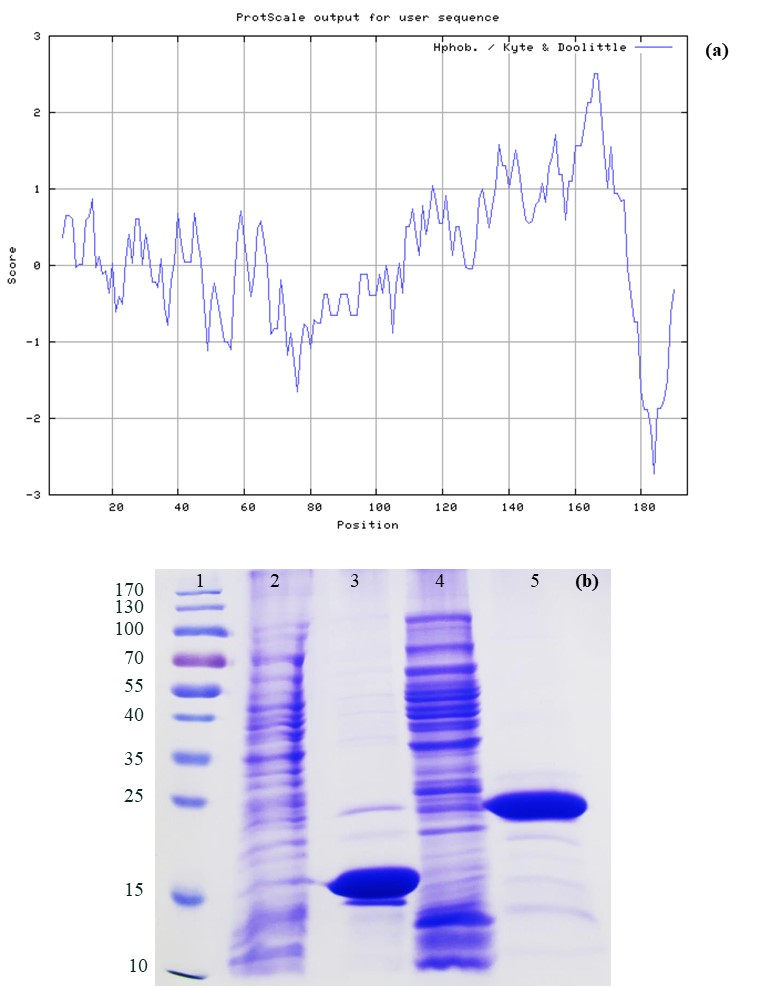


**Figure S1.** (a) Hydrophobicity spectrum of Mms6 protein from *M. marinus* MC-1. (b) SDS-PAGE of purified MamC (lane 3) and Mms6 (lane 5). *E. coli* TOP10 proteins before the purification of MamC (lane 2) and Mms6 (lane 4). Lane 1, molecular weight marker (KDa).


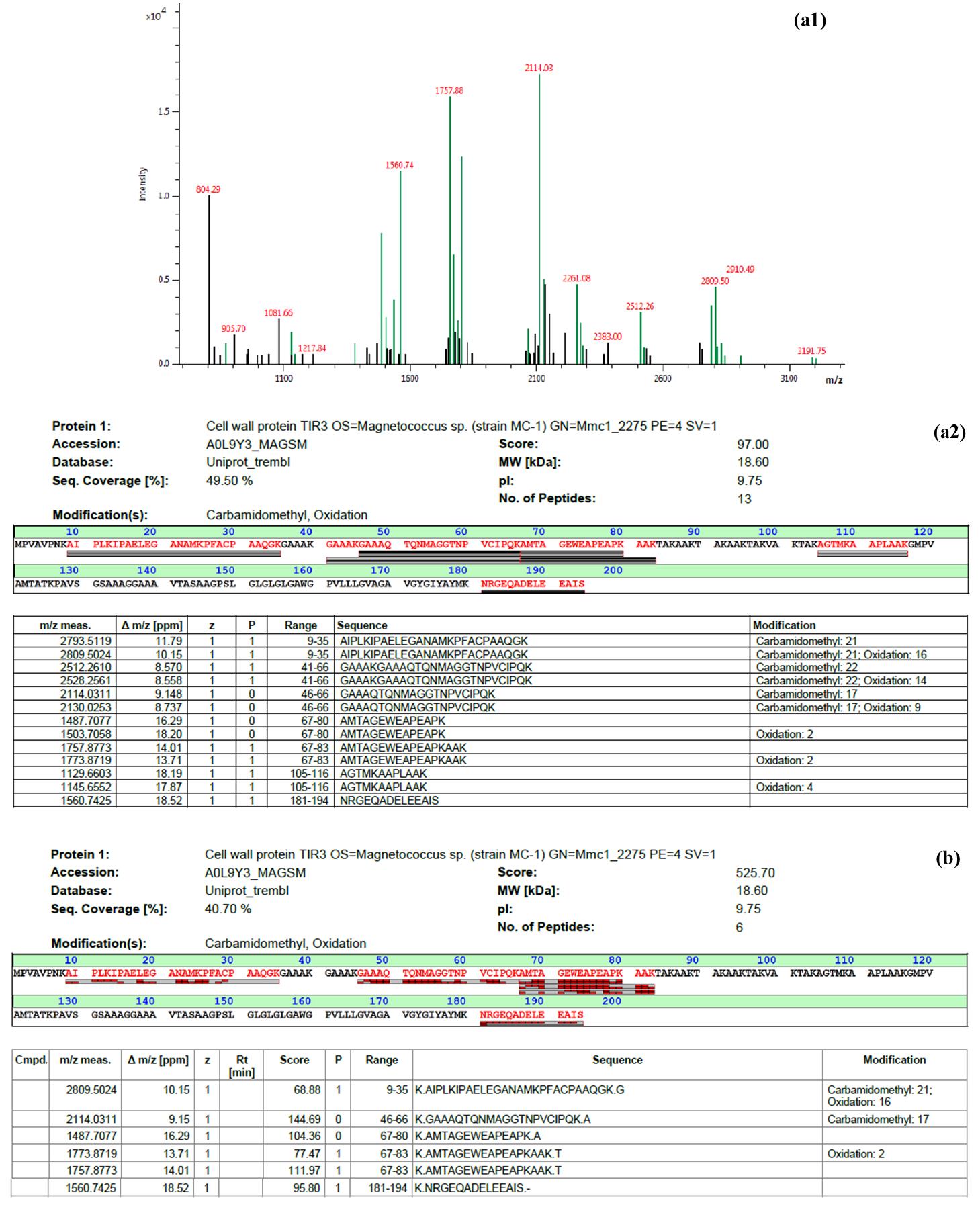


**Figure S2.** (a) Peptide mass fingerprinting (PMF) of Mms6 protein. (a1) Peptide mass and fragmentation spectra, (a2) Peptide spectra comparison result by using MASCOT 2.4.0 (MatrixScience) software^54^ as a search engine. (b) Peptide fragmentation (PFF) by MALDI-TOF/TOF and database comparison by using MASCOT 2.4.0 (MatrixScience) software^54^ as a search engine.

**Table S1.** Prediction of transmembrane regions (SOSUI Program, Nagoya University, Japan).

**
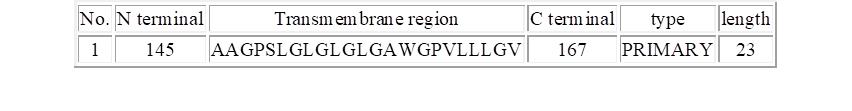
**

**
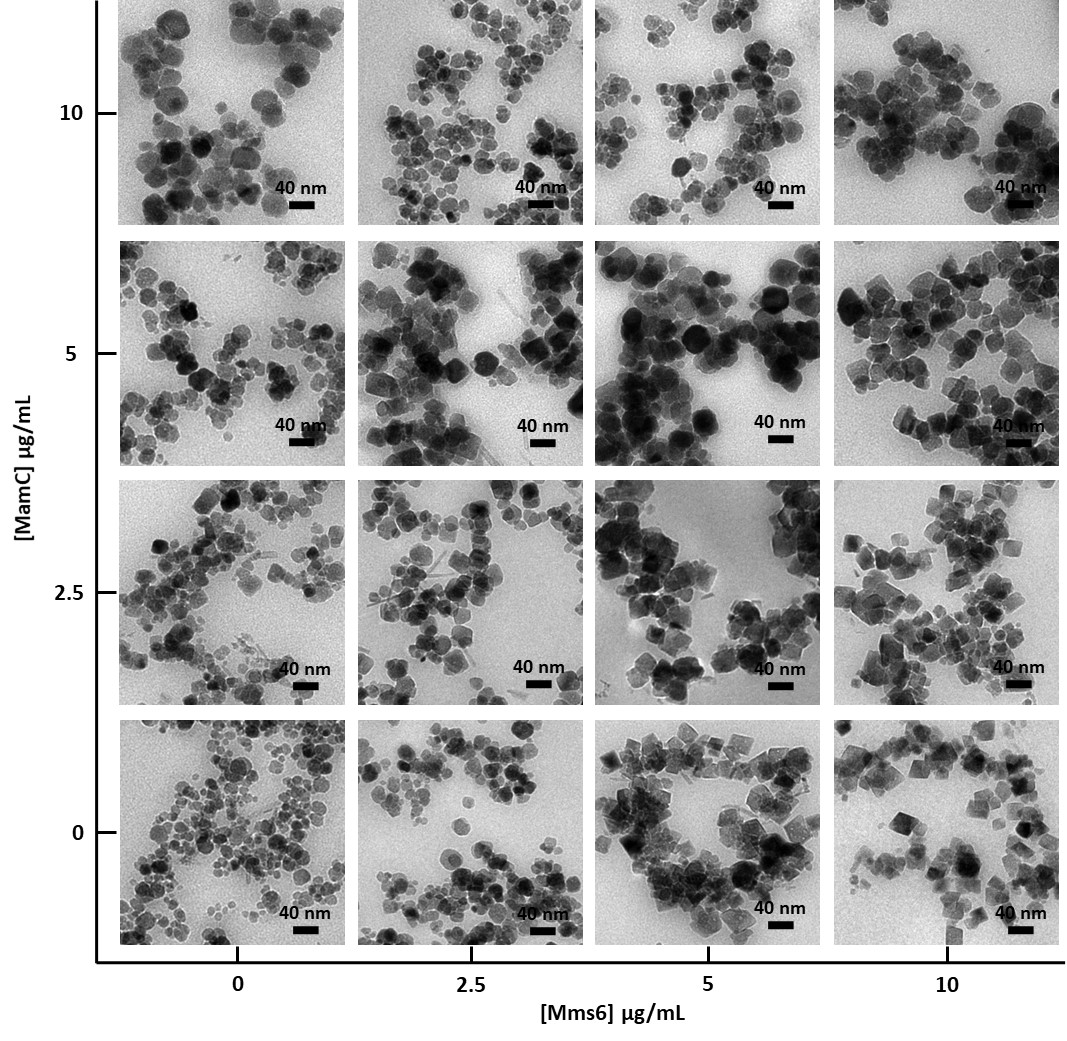
**

**Figure S3.** TEM images of particles obtained in MamC-experiments, Mms6-experiments and Mms6-MamC-experiments.


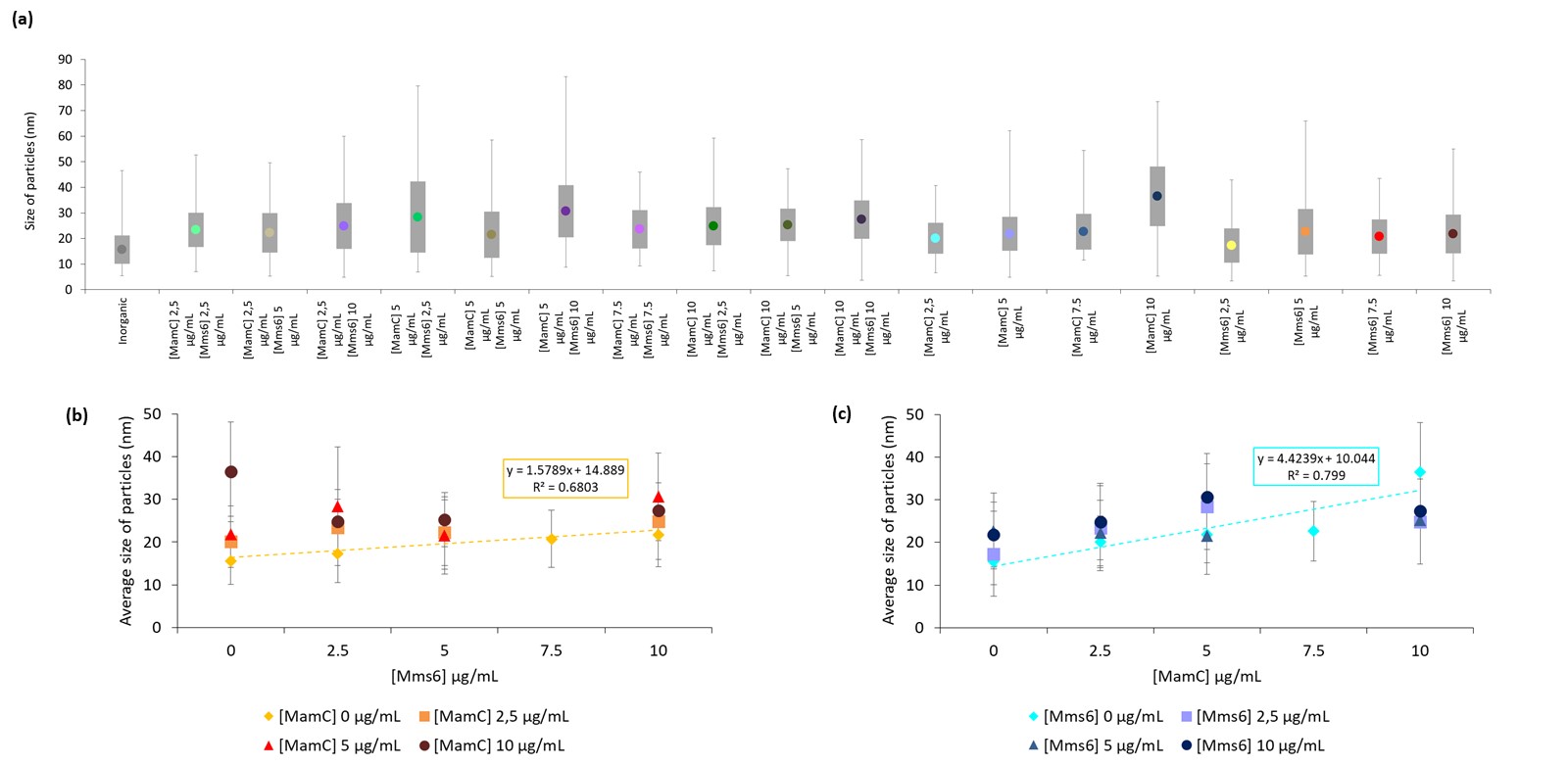


**Figure S4.** (a) Box plot of the size of magnetites obtained in inorganic, MamC-experiments, Mms6-experiments and Mms6-MamC-experiments. Average size of the magnetite crystals obtained in the Mms6-MamC-experiments versus (b) [Mms6] and (c) [MamC].

**Table S2.** ANOVA test of the values of the size averages for all the samples. The difference between averages is significant when α<0.05.

| **Control** | **[MamC]**  **2.5 µg/mL** | **[MamC] 5 µg/mL** | **[MamC] 7.5 µg/mL** | **[MamC] 10 µg/mL** | **[Mms6]**  **2.5 µg/mL** | **[Mms6] 5 µg/mL** | **[Mms6] 7.5 µg/mL** | **[Mms6] 10 µg/mL** | **[MamC]**  **2.5 µg/mL [Mms6]**  **2.5 µg/mL** | **[MamC]**  **2.5 µg/mL [Mms6]**  **5 µg/mL** | **[MamC]**  **2.5 µg/mL [Mms6]**  **10 µg/mL** | **[MamC]**  **5 µg/mL [Mms6]**  **2.5 µg/mL** | **[MamC]**  **5 µg/mL [Mms6]**  **5 µg/mL** | **[MamC] 7.5 µg/mL [Mms6] 7.5 µg/mL** | **[MamC] 5 µg/mL [Mms6] 10 µg/mL** | **[MamC]**  **10 µg/mL [Mms6]**  **2.5 µg/mL** | **[MamC] 10 µg/mL [Mms6] 5 µg/mL** | **[MamC]**  **10 µg/mL [Mms6]**  **10 µg/mL** |
| --- | --- | --- | --- | --- | --- | --- | --- | --- | --- | --- | --- | --- | --- | --- | --- | --- | --- | --- |
| **Control** | 3.33E-16 | 3.33E-16 | 1.66533E-15 | 3.33E-16 | 2.00E-5 | 3.33E-16 | 1.88738E-15 | 3.33E-16 | 1.66E-15 | 1.66E-15 | 1.66E-15 | 1.66E-15 | 1.66E-15 | 1.66533E-15 | 1.66E-15 | 1.66E-15 | 1.66E-15 | 1.66E-15 |
|  | **[MamC] 2.5 µg/mL** | 1.48E-5 | 1.62139E-11 | 3.33E-16 | 6.44E-14 | 3.05E-12 | 0.97712 | 2.65E-5 | 2.55E-15 | 1.18E-6 | 1.66E-15 | 1.66E-15 | 0.0066 | 0.99965 | 1.66E-15 | 1.66E-15 | 1.66E-15 | 1.66E-15 |
|  |  | **[MamC] 5 µg/mL** | 0.37637 | 3.33E-16 | 3.33E-16 | 0.1838 | 0.77915 | 1 | 0.0027 | 0.9880 | 1.27E-13 | 1.66E-15 | 0.9993 | 7.64433E-4 | 1.66E-15 | 1.63E-13 | 1.66E-15 | 1.66E-15 |
|  |  |  | **[MamC] 7.5 µg/mL** | 1.66533E-15 | 1.66533E-15 | 1 | 0,00371 | 0.30331 | 0.68081 | 0,27314 | 3.34601E-7 | 1.66533E-15 | 0.13228 | 1.50655E-10 | 1.66533E-15 | 4.16832E-7 | 2.73252E-10 | 2.22045E-16 |
|  |  |  |  | **[MamC] 10 µg/mL** | 3.33E-16 | 3.33E-16 | 1.66533E-15 | 3.33E-16 | 1.66E-15 | 1.66E-15 | 1.66E-15 | 1.66E-15 | 1.66E-15 | 1.66533E-15 | 1.66E-15 | 1.66E-15 | 1.66E-15 | 1.66E-15 |
|  |  |  |  |  | **[Mms6] 2.5 µg/mL** | 3.33E-16 | 3.37859E-7 | 3.33E-16 | 1.66E-15 | 1.66E-15 | 1.66E-15 | 1.66E-15 | 1.66E-15 | 1.9984E-15 | 1.66E-15 | 1.66E-15 | 1.66E-15 | 1.66E-15 |
|  |  |  |  |  |  | **[Mms6] 5 µg/mL** | 0.05203 | 0.1404 | 0.76535 | 0.98429 | 1.04E-6 | 1.66E-15 | 0.11529 | 8.09948E-10 | 1.66E-15 | 1.29E-6 | 1.19E-9 | 1.66E-15 |
|  |  |  |  |  |  |  | **[Mms6] 7.5 µg/mL** | 0.8176 | 0.00416 | 0,0116 | 3.11908E-8 | 1.66533E-15 | 0.98199 | 0.86255 | 1.66533E-15 | 3.67462E-8 | 4.55078E-10 | 2.22045E-16 |
|  |  |  |  |  |  |  |  | **[Mms6] 10 µg/mL** | 0.00202 | 0.97808 | 9.49E-14 | 1.66E-15 | 0.99981 | 0.00126 | 1.66E-15 | 1.20E-13 | 1.66E-15 | 1.66E-15 |
|  |  |  |  |  |  |  |  |  | **[MamC] 2.5 µg/mL [Mms6] 2.5 µg/mL** | 0.092 | 0.00296 | 1.66E-15 | 8.11E-5 | 1.13354E-13 | 1.66E-15 | 0.00354 | 1.76E-5 | 1.66E-15 |
|  |  |  |  |  |  |  |  |  |  | **[MamC] 2.5 µg/mL [Mms6] 5 µg/mL** | 2.24E-10 | 1.66E-15 | 0.724 | 2.91037E-5 | 1.66E-15 | 2.85E-10 | 4.7E-14 | 1.66E-15 |
|  |  |  |  |  |  |  |  |  |  |  | **[MamC] 2.5 µg/mL [Mms6] 10 µg/mL** | 1.66E-15 | 1.66E-15 | 1.66533E-15 | 1.66E-15 | 1 | 0.986 | 3.73E-9 |
|  |  |  |  |  |  |  |  |  |  |  |  | **[MamC] 5 µg/mL [Mms6] 2.5 µg/mL** | 1.66E-15 | 1.66533E-15 | 2.74E-7 | 1.66E-15 | 9.79E-14 | 0.241 |
|  |  |  |  |  |  |  |  |  |  |  |  |  | **[MamC] 5 µg/mL [Mms6] 5 µg/mL** | 0.05073 | 1.66E-15 | 1.77E-15 | 1.66E-15 | 1.66E-15 |
|  |  |  |  |  |  |  |  |  |  |  |  |  |  | **[MamC] 7.5 µg/mL [Mms6] 7.5 µg/mL** | 1.66533E-15 | 1.66533E-15 | 1.66533E-15 | 2.22045E-16 |
|  |  |  |  |  |  |  |  |  |  |  |  |  |  |  | **[MamC] 5 µg/mL [Mms6] 10 µg/mL** | 1.66E-15 | 1.66E-15 | 2.88E-15 |
|  |  |  |  |  |  |  |  |  |  |  |  |  |  |  |  | **[MamC] 10 µg/mL [Mms6] 2.5 µg/mL** | 0.98 | 2.21E-9 |
|  |  |  |  |  |  |  |  |  |  |  |  |  |  |  |  |  | **[MamC] 10 µg/mL [Mms6] 5 µg/mL** | 2.43E-6 |
|  |  |  |  |  |  |  |  |  |  |  |  |  |  |  |  |  |  | **[MamC] 10 µg/mL [Mms6] 10 µg/mL** |

**Methods**

**Analysis of Mms6-MamC interaction**

The interaction between Mms6 and MamC has been investigated by using AlphaScreen assays. MamC is captured by a monoclonal mouse anti-MamC antibody (ProteoGenix, 1 mg/mL in PBS buffer added with 0.02% sodium azide), which will itself bind to anti-mouse IgG Acceptor beads (Perkin Elmer Cat. 6760606C), while Mms6 is captured by a polyclonal rabbit anti-Msm6 antibody (target sequence Cys_YMKNRGEQADELEE, ProteoGenix, 0.84 mg/mL in PBS buffer added with 0.02% sodium azide), which will itself bind to anti-rabbit IgG Donor beads (Perkin Elmer Cat. AS105D). When Mms6 and MamC are interacting, the Donor and Acceptor beads come into proximity. Excitation of the Donor beads results in emission of light from the Acceptor beads. The signal generated is proportional to the amount of proteins that are interacting. This test was chosen to study the interaction between Mms6 and MamC because it offers advantages over other assay formats as they do not require any wash steps, have fewer protocol steps, require less sample, and provide a large dynamic range. A stock solution of Msm6 (1 mg/ml) in H_2_O with Triton X-100 and of MamC (1 mg/ml) in 50 mM Tris, 150 nM NaCl, pH 8 were used in the experiment and diluted by adding the here referred as assay buffer (50 mM NaH_2_PO_4_, pH 8, 150 mM NaCl, 0.05% Tween-20, 0.1% BSA) to reach the concentrations in Figure S5. An experiment was performed to titrate each protein in a cross titration matrix on the half-area of 96-well Optiplate (Figure S5) in order to find their optimal concentration in the assay. Titration of one protein down the plate and the other protein across the plate allows a view of all the combinations and includes the important controls of no proteins and single protein. Mms6 and MamC proteins at the specific concentration were added to the well and allowed to interact for 60 minutes at 28 ºC. Experiments with only MamC or Mms6 and with no proteins were used as negative controls. Then after, anti-Mms6, anti-MamC, and acceptor and donor beads were added to all the wells and let to react for 120 minutes at 23 ºC.The final concentration of the anti-Mms6, anti-MamC, and acceptor and donor beads were of 1 nM, 3nM and 20 μg/mL, respectively. Finally the signal was read by using EnsightTM Perkin-Elmer.


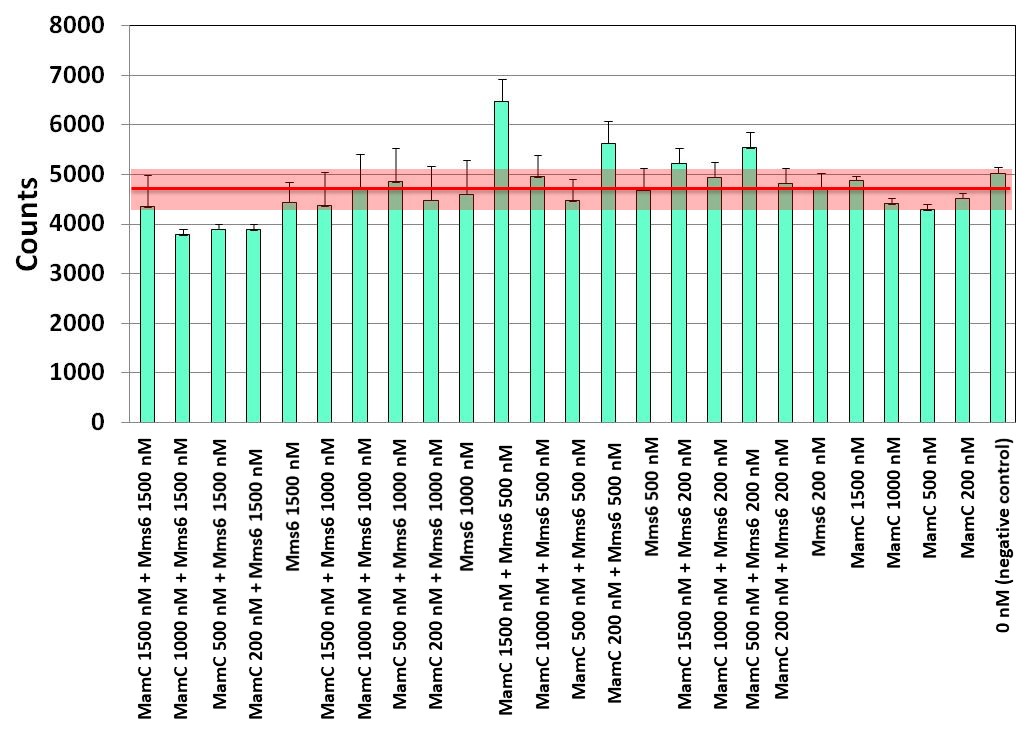


**Figure S5.** AlphaScreen assay of the Mms6-MamC interaction indicating the concentration of each protein used and the total counts obtained for the different proteins concentration interaction.
